# The health disparities research industrial complex as structural violence: insights from the U.S. South

**DOI:** 10.3389/fpubh.2026.1870761

**Published:** 2026-07-13

**Authors:** Opal Gay, Kirsten Cook

**Affiliations:** Partnership for Southern Equity, Atlanta, GA, United States

**Keywords:** community-based research, epistemic violence, health disparities, racial health equity, racial equity, structural racism, U.S. South

## Abstract

The U.S. South experiences some of the most severe and persistent health inequities in the nation, shaped by a long history of racialized structural violence in housing, healthcare, education, and the criminal legal system. At the same time, the region remains a critical site of resistance, rooted in deep traditions of community organizing that have reshaped national understandings of social injustice. Against this backdrop, the Partnership for Southern Equity launched its Community-Informed Research for Change, Learning, and Equity (CIRCLE) initiative to address an enduring and underexamined form of structural discrimination: the unequal control of research, data, and knowledge production in communities of color. Drawing on CIRCLE’s ongoing landscape scan, this article interrogates the Health Disparities Research Industrial Complex (HRIC) as a site of structural violence. This article draws on the Black radical tradition of the U.S. South, recognizing the control of knowledge as historically inseparable from the project of white oppression. Situated in a region bearing the sharpest edge of racialized health inequities, while also holding the nation’s deepest traditions of community-rooted resistance, this article names the mechanisms through which epistemic violence operates in the present and uplifts the community-driven strategies already emerging across the South as pathways toward a more equitable and accountable system of knowledge production.

## 1 Introduction

For all its complex and troubled history, the U.S. South is also a place of hope and opportunity, where the growth of the region’s racial diversity is met with promising expressions of the Third Reconstruction, as coined by historian Joseph (1). More than half of Black Americans live in the South, and the region has the fastest-growing Latino and Asian populations in the United States (2). Amidst the backdrop of a federal political regime steeped in racism and intent on undermining civil rights protections, the South is also seeing “some of the most innovative social justice campaigns in the country” (3). Yet, the South’s ingenuity is far from novel. Communities across the region encounter the sharpest and most sustained edge of institutionalized white oppression, a legacy that persists within contemporary concentrations of restrictive voting laws and the most severe deprivation of health care coverage in the nation (4, 5). Such conditions have mandated an orientation towards building power beyond hegemonic institutions.

One such arena of promising change, and the highlight of this paper, is the project of naming, resisting, and providing alternatives to the systems and structures through which knowledge is produced, validated, and disseminated. Ezell (6) terms this structural domain the Health Disparities Research Industrial Complex (HRIC), a self-perpetuating system of epistemological violence in which (a) institutional arrangements sustain minority health disadvantage, (b) research authority is concentrated among those without lived connection to affected communities, and (c) scholarship remains structurally incapable of addressing the inequities it documents. In the American context, race has been a central axis through which the HRIC’s three mechanisms have operated and compounded. The United States was built, in no small part, on the manipulation of knowledge to institutionalize white oppression (7). Settler colonial occupation, the attempted genocide of Indigenous peoples, and the institution of chattel slavery were actively legitimized and sustained by an emerging body of European scientific knowledge that, from the 15th century onward, marshaled tools of Enlightenment reason to naturalize racial hierarchy and rationalize mass levels of violence (8). The legacy of white oppression within American science did not end with emancipation, Reconstruction, or the civil rights victories of the 20th century, but continues well into the present day. Nowhere is this dynamic more apparent than the U.S South, where the legal entrenchment of Black people as second-class citizens created the conditions for some of the most egregious violations of research ethics in American history. That 75% of Black adults report awareness of the Tuskegee syphilis study and 68% recall eugenic sterilization projects speaks to the enduring weight of racist scientific practice, and the conditions from which the HRIC continues to plague communities of color (9).

Mirroring this history, however, revolutionary community institutions, organizers, and researchers have explored transformational methodologies and practices, leading to necessary and exciting mindset shifts in the field. These efforts have deep roots in the U.S. South, where generations of Black communities have built knowledge systems, organizational infrastructure, and research traditions outside of and often in resistance to dominant institutions—from the Freedom Schools of the Civil Rights Movement to the community health clinics of the Black Panther Party to the contemporary networks of community-of-color-led nonprofits and advocacy organizations that continue to document, analyze, and respond to racial inequities on their own terms. It is from this fertile ground that the Partnership for Southern Equity launched the ‘Community-Informed Research for Change, Learning, and Equity’ (CIRCLE) initiative, to organize, connect, and amplify the collective power of our region at a scale this political moment requires.

CIRCLE emerged from a recognition that researchers and communities of color across the U.S. South have long been producing liberatory knowledge, recounting subjugated histories, and resisting epistemological violence, often with little institutional support and against systems structured to protect the status quo. This long-term initiative seeks to resist problematic assumptions behind mainstream data and research practices, as well as facilitate partnerships and a community-led ecosystem that shifts research authority back towards communities whose wisdom has been structurally excluded from the production of knowledge. Currently in Phase Zero, a deliberate period of systematic observation and expert engagement, CIRCLE’s landscape scan is examining how epistemological violence operates across each arena of the Health Disparities Research Industrial Complex in the U.S. South. Drawing on insights from Phase Zero, including dozens of conversations held with experts, researchers, practitioners, funders, and community leaders, this article reports where oppression derived from the HRIC persist most acutely, and uplifts the community-driven strategies that offer promising pathways toward a more equitable system for producing knowledge.

## 2 Institutionalizing white oppression

Which institutions receive resources and whose knowledge is granted legitimacy constitute the basis of what makes racial health equity possible at scale. At a technical level, the U.S. university system still functions as the primary arbiter of research legitimacy—controlling credentialing systems, peer review processes, and the publication infrastructures through which findings gain institutional recognition and policy influence. However, universities are also deeply entangled with state and federal government bureaucracies in ways that make their research agendas inherently political. When government aid flows to universities, institutions respond in ways that serve their own revenue and survival interests, often reproducing or deepening the inequalities the funding was ostensibly designed to address (10, 11). This structural dependency makes universities, and by extension the research they produce, subject to the shifting priorities of neoliberal political agendas.

For example, the change taking place across the U.S. university system under the current administration is staggering. By declaring racial and health equity initiatives discriminatory under civil rights law, the Trump administration has turned the legal architecture built to dismantle racial segregation into a tool for defunding the programs designed to address its consequences. The deeply partisan Supreme Court has effectively co-signed, ruling 5–4 to allow $783 million in National Institutes of Health research cuts to proceed (12). The ruling is part of a broader wave of terminations that has seen $2.2 billion cut from Harvard alone and hundreds of grants targeting health equity in rural and communities of color terminated outright (13). The Offices of Minority Health under both Centers for Medicare and Medicaid Services and the Department of Health and Human Services have been gutted, the Department of Justice has declared health equity funding discriminatory, and more than 50 universities are under federal investigation, with state and local health officials declining to speak publicly for fear of retaliation (14, 15). This assault extends to the state and local level. CRT Forward, an initiative out of UCLA School of Law that tracks legal measures restricting truthful discussion of race and systemic racism, found that every state in the U.S. South has adopted at least one such measure at the state level (16). Among the 47 Southern R1 universities our project has tracked since Trump’s 2024 election, only two retained existing DEI policies without documented changes, while most either rolled back DEI infrastructure or renamed diversity offices in ways that may obscure continued equity work under less politically contested language.

Philanthropy has not filled the gap. Despite the brief nationwide racial reckoning of 2020 that led to, in many cases, short-lived and even sometimes opportunistic philanthropic and corporate financial commitments, the South still receives less than 3% of philanthropic dollars nationally, and Black-led nonprofits remain chronically underfunded and excluded from the sustained funder relationships that build research capacity over time (17). White oppression has long been embedded in philanthropic structures, shaping not only who receives funding but on whose terms. Rooted in what Scott et al. (18) call “philanthropic redlining,” the inequitable distribution of resources away from Black-led organizations reflects discriminatory assumptions about their capacity and legitimacy. James-Gallaway (19) demonstrates that white philanthropists have historically supported Black communities primarily when doing so served white economic and political interests, a pattern that Kraeger (20) argues persists today when philanthropy substitutes transactional grantmaking for genuine community accountability. Compounding this, casting Black communities as recipients rather than philanthropic agents in their own right has entrenched a white-savior narrative that obscures the self-determined institution-building that has long sustained Black life where external support has failed (21).

## 3 Concentrating white authority

Determining “who” gets to conduct research in the health science fields is an essential function of the Health Disparities Research Industrial Complex. This gatekeeping mechanism operates primarily through academic institutions, where the price of admission has done much of the work of exclusion. In a society where wealth and whiteness remain deeply correlated, high tuition costs have produced a research workforce that reflects who can afford to enter the field rather than who is most affected by the disparities it studies (6). Over the past two decades, health science programs have expanded dramatically, with the number of accredited schools of public health tripling in the past 15 years, and medical school applications grew by nearly 70% between 2000 and 2022 (51). During this same period, annual tuition at top research institutions has climbed well into the tens of thousands of dollars, a burden borne disproportionately by students from underrepresented racial backgrounds. Residents of the U.S. South currently hold well over $600 billion in collective student loan debt, with Black college graduates owing, on average, 188% more than their white counterparts after graduation (48, 49). This dynamic not only suppresses the entry of Black researchers into the pipeline but saddles those who do persist with debt loads that make underpaid community-based work untenable upon graduation (6).

Racial and ethnic minority graduates remain systematically underrepresented across accredited schools and programs of public health relative to the populations those institutions serve, a disparity that sharpens at higher degree levels and is most acute in doctoral programs (22). First-generation students of color who do navigate the pipeline into public health careers face additional compounding burdens along the way: financial fragility, the labor of navigating institutional hidden curricula without familial guidance, and the chronic absence of mentors with shared identities and experiences (23). The composition of the faculty training the next generation of researchers compounds these disparities further. Among 543 public four-year institutions examined in 2020, more than half received failing grades for Black faculty diversity relative to their own student enrollment, and Black and Latino faculty were disproportionately concentrated in contingent rather than tenure-track appointments, the positions that carry the mentorship capacity and research influence most critical to pipeline change (24).

What modest progress had been made toward diversifying the field is now being actively reversed under the Trump administration. The share of Black and Hispanic faculty at top research universities grew from 9 to 12% between 2015 and 2024 following a wave of institutional commitments, but most of those programs are now frozen or abandoned as the federal government withholds funding and investigates university diversity initiatives (25). Following a federal investigation, more than 31 major research universities severed ties with the PhD Project, a nonprofit that had helped more than 1,500 racial minority scholars earn doctoral degrees, after the Department of Education deemed such support a violation of civil rights law (26). The Supreme Court’s 2023 elimination of race-conscious admissions and sweeping DEI restrictions across Southern states have compounded an already narrowed pathway (27, 46).

The concentration of research authority among white professionals extends beyond the academy into the governmental agencies, health departments, and nonprofit organizations where much of the applied and community-based research training and practice in the South actually takes place. People of color represent only 42% of governmental public health employees nationally, and those who are present are concentrated disproportionately in administrative and clerical positions rather than the scientific and leadership roles with the greatest capacity to shape research agendas (28). Only 60% of local health departments are representative of the communities of color they serve, with rural and Southern departments showing the greatest gaps (29, 30). The nonprofit health sector tells the same story: white professionals hold 77% of leadership roles, and white-led organizations command 5.5 times as much sector-wide revenue as those led by people of color, a gap that directly constrains the capacity of community-rooted organizations to conduct and sustain research (31). The South’s rich racial and ethnic diversity and deep tradition of community-rooted organizing are reflected in the fact that Georgia, Louisiana, and Mississippi rank among the states with the highest rates of Black, Indigenous, and People of Color nonprofit board representation nationally, yet these same organizations are often among the most chronically underfunded (31).

## 4 Scholarship incapable of advancing racial health equity

Historically, the methods deemed legitimate by academic institutions and peer reviewers have reflected the standards and values of white oppression standards and values (32). Still, to this date, generally accepted and “standard” practices for data collection, analysis, interpretation, and communication in the health sciences have had both intentional and unintentional harmful impacts on communities of color. Persisting distrust among communities of color toward research institutions reflects something larger than historical memory or individual encounters with prejudice; it is an indicator of how racism continues to operate through the systems and standards that govern knowledge production (32–34, 44, 45, 47). Quantitative methods continue to be taught and practiced as the dominant standard of rigor, presented as objective even as critical scholars have documented how routine methodological choices reproduce inequity and carry those assumptions forward into publications, grants, and training (35). Comparing groups against dominant reference categories, excluding outliers, dismissing small samples, and treating nonresponse as a technical flaw are common practices that can produce deficit-based understandings of minoritized communities while appearing methodologically sound, obscuring the structural conditions that generate harm in the first place (35). As well, in their critical extension of the HRIC framework, Mrig and Spencer (36) argue that the hegemony of quantitative methods has structurally devalued qualitative inquiry, foreclosing knowledge of the processes through which structural racism shapes health.

Community-engaged work often requires more time, deeper trust, and more resource-intensive relationship building, yet academic institutions continue to privilege conventional measures of success tied to peer-reviewed publication, disciplinary prestige, and tenure expectations, making slower and more relational forms of inquiry harder to sustain (37). That helps explain why participatory approaches may be praised in theory while power and decision making still remain weighted toward academic partners in practice, with participation sometimes functioning less as shared governance than as a means of accessing community knowledge while leaving underlying asymmetries of power intact (38, 39). Even as community members supply access, trust, labor, and lived knowledge, academic researchers—often who are not themselves part of the communities being studied—still retain final authority over what gets asked, how evidence is interpreted, and what gets published (37).

Beyond how research is designed, the knowledge produced largely remains inaccessible to the communities most affected by the inequities in question. Academic and medical researchers still largely write for peer reviewers and evaluate their success through publication, citation, and funding metrics rather than community usefulness, which makes community engaged work harder to sustain institutionally (37). As well, the findings communities most need are routinely presented in formats that are text and jargon heavy, densely written, or locked behind paywalls that require institutional access (40). Under these conditions, communities affected by health inequities are often positioned as sources of data rather than the primary audience research is accountable to, which can weaken translation, deepen mistrust, and limit the practical value of findings (38).

## 5 Discussion

The Health Disparities Research Industrial Complex is a social system with roots deeper than the origins of this nation. The concentration of white oppression within the institutional arrangements, positions of authority, and practices that govern knowledge production trace directly to a history in which science has actively suppressed the social mobility of communities of color. The tools of dominant research may allow scholars to document inequities, secure funding, publish findings, and briefly challenge the terms of debate, but, as Lorde (50) famously warned, “the master’s tools will never dismantle the master’s house.” If health disparities research is governed by the same universities, funders, review processes, and professional hierarchies that have historically flattened racial health inequities into empirical data while leaving the organization of power intact, then the path forward cannot be limited to reforming the master’s house from within. It must also require imagining forms of intellectual, political, and institutional autonomy capable of producing knowledge outside the terms of government, corporations, and university.

Across the U.S. South, Black communities and racial equity organizations are already building the kind of alternative knowledge infrastructures that reflect what community-sufficient research looks like in practice. The Partnership for Southern Equity’s Metro Atlanta Equity Atlas offers just one prominent example of a Southern organization democratizing access to regional and neighborhood-level data for advocacy, planning, community power, and tracking local conditions over time. These efforts represent one piece of a broader shift toward community-controlled research infrastructure, a shift that also requires new mechanisms for determining who has the authority to approve, evaluate, and legitimate research. The Beloved Community Institutional Review Board offers one such mechanism, shifting research oversight away from the university as the assumed center of ethical authority. Housed at a Black womxn-led nonprofit in New Orleans, the federally recognized IRB was created to support people-centered research, reclaim knowledge from universities, and disrupt a culture of white oppression in research production and dissemination. More broadly, collectives such as Data for Black Lives have begun building national networks of scientists and activists committed to challenging discriminatory data practices, crowdsourcing funds for liberatory projects, and redistributing resources directly to the grassroots organizations doing community-based research.

Importantly, Ezell (6) reminds us that any effort to disrupt the HRIC must include expanding who is able to become a researcher within our current system. This means preserving equity in recruitment and admissions, even in a politically restrictive climate. The Southern Regional Education Board offers an important Southern-rooted model for building that pipeline through sustained financial, professional, and early-career support for underrepresented Ph.D. students. Since 1993, its State Doctoral Scholars Program has supported more than 2,300 scholars across 113 institutions in more than 30 states. Its reach has been especially significant for African American scholars, who make up the overwhelming majority of participants (74%), alongside Hispanic American, Asian American, American Indian and Alaska Native, and other scholars historically underrepresented in doctoral education. Yet a major gap not only remains but is growing. The sunsetting of similar support programs, such as the Ford Foundation Fellowship and the Robert Wood Johnson Foundation’s Health Policy Research Scholar program, marks a significant loss in the national infrastructure matriculating underrepresented researchers into the field. For equity-driven investors, this moment calls for renewed commitments, as well as cultivation of new programs that allow students of color, queer students, disabled students, first-generation students, and immigrant students to complete their degrees and enter health equity research with dignity. We additionally amplify existing calls from critical race scholars, including Ameen et al. (41), who argue that systems-level change requires building the research capacity of Predominantly Black Institutions and Historically Black Colleges and Universities, while strengthening pathways that support Black people’s entry into health research from K-12 education through academic leadership. Such pathways require early exposure to health research careers, sustained undergraduate and graduate training, mentorship, professional development, and targeted efforts to close funding gaps for Black principal investigators leading racial health equity research.

In general, there is a compelling need to diversify funding sources for genuine and grassroots community work. Philanthropy, especially, must systematically transform how it distributes money, authority, and institutional legitimacy. We cosign the argument by Scott et al. (18) that funders should be internally audited for racial justice assessments, preferably by third-party community-of-color-led organizations and researchers. We additionally recommend Kraeger’s (20) call for philanthropy to move beyond symbolic listening by using deliberative practices, such as citizens’ assemblies, mini-publics, deliberative polls, and participatory grantmaking, to let communities shape funding and policy agendas. Rather than asking how philanthropy can protect itself from political backlash, the more urgent question is how funders can materially defend the communities, organizations, and knowledge infrastructures they claim to support. The 2024 Diversity Among Philanthropic Professionals report suggests that, prior to the 2024 presidential election, diversity appeared to be increasing among participating foundations, with people of color comprising 54.8% of respondents in 2024, compared with 43.2% in 2022. Yet the larger Council on Foundations survey, which found that people of color comprised only 33.5% of the philanthropic workforce in 2024, a 21-point gap, points to a more polarized sector in which equity-oriented foundations are more willing to participate in diversity assessment while less diverse institutions remain less accountable (42). Within the current political climate, it is especially important for philanthropic actors to heed the report’s call to “be bold and speak out” against policies aimed at erasing diversity, equity and inclusion and civil rights [(42), p. 17]. A more accountable and reparative model, especially within the U.S. South, would invest in Black-led institutions, community-controlled research infrastructure, and Black philanthropic traditions, including giving circles and Black-created foundations that position Black communities as builders of knowledge, resources, and social transformation rather than as passive recipients of aid (21).

Lastly, disrupting the HRIC requires transforming how racial health equity research is designed and disseminated to the communities from which it is derived. Ultimately, achieving this transformation requires treating liberatory, community-based participatory research as the standard for racial health equity science (34). This means moving beyond inaccessible academic frameworks and toward creative, narrative, and reflexive methods that honor community wisdom and culture as legitimate sources of knowledge. Additionally, community participation and leadership illuminate structural conditions in ways outside perspectives cannot, opening pathways toward structural solutions (34, 38). As Bowleg (43) argues, academics have long prioritized producing ever more documentation of racial health inequities while investing comparatively little in the interventions capable of transforming them. As she succinctly argues, genuine disruption to the white racial frame will require moving beyond system-blind research on population-level health disparities that emphasize individual-level health behaviors. In its place must emerge a pragmatic scientific culture defined by sustained investment in structural analysis, researcher reflexivity, and the centering of community-rooted innovations that address racial health inequities at their source rather than documenting their symptoms.

Mrig and Spencer (36) caution, however, that calling on researchers to embrace community-based participatory approaches without first disrupting the conditions that penalize such work risks reproducing the very dynamics of the HRIC it seeks to dismantle. Institutional transformations such as those outlined above are therefore the necessary foundation upon which genuine methodological change can take root. As well, academic research departments should move toward institutionalizing community-co-designed curricula and programming, so that future scholars are equipped to serve community-defined priorities rather than impose externally driven agendas. Access to existing research infrastructure matters here as well. Many public universities already grant some level of community access to institutional libraries and research resources, but such efforts remain limited and uneven. As publicly funded institutions, universities have an unrealized obligation to open their research infrastructure to the communities their work purports to serve, including, but not limited to, local residents, youth, artists, business leaders, independent researchers, community organizers and advocates, public defenders, and policy practitioners.

## 6 Conclusion

The Health Disparities Research Industrial Complex is not simply a flawed research ecosystem, but an enduring site of white supremacist structural violence. The HRIC extracts data from communities of color while limiting their access to the same institutions, resources, and authorities that govern knowledge production. Disrupting this system requires more than better engagement practices, diversity statements, or inclusive research teams, though each may matter in the short term. Such a transformation requires changing the institutional conditions that determine how racial health equity research is produced and who it ultimately serves. The path ahead is long, but it begins by refusing to treat the current system as normal or fixed. As the current presidential administration exposes the fragility of institutional commitments to racial equity, the urgent task is to recognize, resource, and collectively mobilize community-driven forms of resistance and innovation wherever they have existed, are taking shape, and continue to emerge. Through the Partnership for Southern Equity’s Community Informed Research for Change, Learning, and Equity initiative, we aim to contribute to this work by building a Southern research and learning infrastructure that connects organizers, researchers, funders, and practitioners already advancing more democratic and community-sufficient models of knowledge production. Our hope is to strengthen the conditions under which communities can shape research questions, govern data, define impact, and access knowledge in service of their own priorities. We imagine a landscape where racial health equity research no longer extracts community suffering for institutional value but helps build the power communities need to determine their own futures.
